# Primary Hepatic Lymphoma Masquerading as Symptomatic Hypercalcemia

**DOI:** 10.7759/cureus.10633

**Published:** 2020-09-24

**Authors:** Abdulla M. Ali, Ibrahim Omore, Muhammad Faisal Riaz, Maria Paliou, Karen Simon

**Affiliations:** 1 Internal Medicine, Harlem Hospital Center, New York, USA; 2 Internal Medicine/ Endocrinology, Harlem Hospital Center, Columbia University College of Physicians and Surgeons, New York, USA

**Keywords:** primary hepatic lymphoma, hypercalcemia, liver biopsy

## Abstract

Primary lymphoma of the liver is extremely rare, and is more common among immunocompromised patients. It typically occurs after the fifth decade of life and has a male predominance. It often presents with diagnostic difficulties to both clinicians and pathologists as most cases have a solitary or multiple mass lesions in the liver with normal alpha-fetoprotein levels. Chemotherapy is the standard of therapy. Here, we describe a unique case of primary hepatic lymphoma in an elderly immunocompetent female who presented with symptomatic hypercalcemia.

## Introduction

Primary hepatic lymphoma (PHL), a type of non-Hodgkin’s lymphoma, is a very rare malignancy that can be mistaken for hepatocellular cancer [1]. Distinction between the two is important because PHL responds to chemotherapy and has a relatively better prognosis. We present a case of PHL in a patient who presented with symptomatic hypercalcemia. Liver is major reticuloendothelial organ and can be involved by secondary systemic non-Hodgkin's lymphoma. Diagnosis of PHL depends on biopsy consistent with lymphoma, and there is no extrahepatic lymphoproliferative involvement.

## Case presentation

An 82-year-old woman presented to our hospital’s emergency department with new onset confusion. Her past medical history includes type 2 diabetes mellitus, hypertension, hyperlipidemia, and age-related osteoporosis on intravenous (IV) bisphosphonate yearly.

Six weeks prior to this admission, she was treated for pyelonephritis at a different hospital. At that time, laboratories were notable for a total calcium of 12.8 mg/dl (ref range, 8.4-10.5 mg/dl), albumin 3.6 g/dl (ref range, 3.5-5.3 g/dl), creatinine 1.6 mg/dl (ref range, 0.50-0.90 mg/dl), phosphorus of 2.7 mg/dl (ref range, 2.5-4.5 mg/dl), alkaline phosphatase (ALP) of 150 U/L (ref range, 35-105 U/L), parathyroid hormone (PTH) intact of 7 pg/ml (ref range, 15-65 pg/ml), and 25-hydroxy vitamin D level of 28.1 ng/ml (ref range 30-80 ng/ml). A 1,25-dihydroxy vitamin D level was not checked. CT of the abdomen without contrast revealed fatty infiltration predominantly of the right lobe of the liver and two hypodense lesions, one at hepatic segment V (largest diameter of 8 cm) and another at hepatic segment VI (largest diameter of 3.4 cm). On the day of discharge, she had a calcium level of 10.6 mg/dl and a creatinine of 0.89 mg/dl. She was discharged with oral ciprofloxacin and 800 units of cholecalciferol daily in addition to her regular outpatient medications.

On presentation to our hospital, her initial vitals were temperature 98.3˚F, heart rate of 81 beats/minute, respiratory rate of 18 cycles/minute, blood pressure of 142/65 mmHg, and oxygen saturation of 100% on ambient air. Physical examination was notable for a cachectic, non-toxic appearing woman with altered mentation. Otherwise, physical examination was unremarkable. Initial laboratory data were notable for a blood urea nitrogen (BUN) of 19 mg/dl, creatinine of 1.5 mg/dl, calcium level of 16.7 mg/dl, albumin of 3.4 g/dl ref (3.9-4.9 g/dl), hemoglobin of 9 g/dl, mean corpuscular volume (MCV) of 79 fl, and ALP of 175 U/L. Bilirubin, aspartate transaminase (AST), and alanine aminotransferase (ALT) were normal. CT head was negative for any acute pathology.

A work-up for hypercalcemia showed a low serum intact PTH of 8 pg/ml (ref range 15-65 pg/dl) and a low parathyroid hormone related peptide (PTHrP) of <2 pmol/L. 1,25 dihydroxy vitamin D was elevated at 81 pg/ml (ref range 19.9-79.3 pg/dl). The patient was admitted and treated for vitamin D dependent hypercalcemia with IV 0.9% sodium chloride infusion, subcutaneous calcitonin, and zoledronate infusion. Calcium levels were closely monitored and gradually trended down. Serum immunofixation did not identify a monoclonal band, and both serum and urine protein electrophoresis were normal.

**Figure 1:**
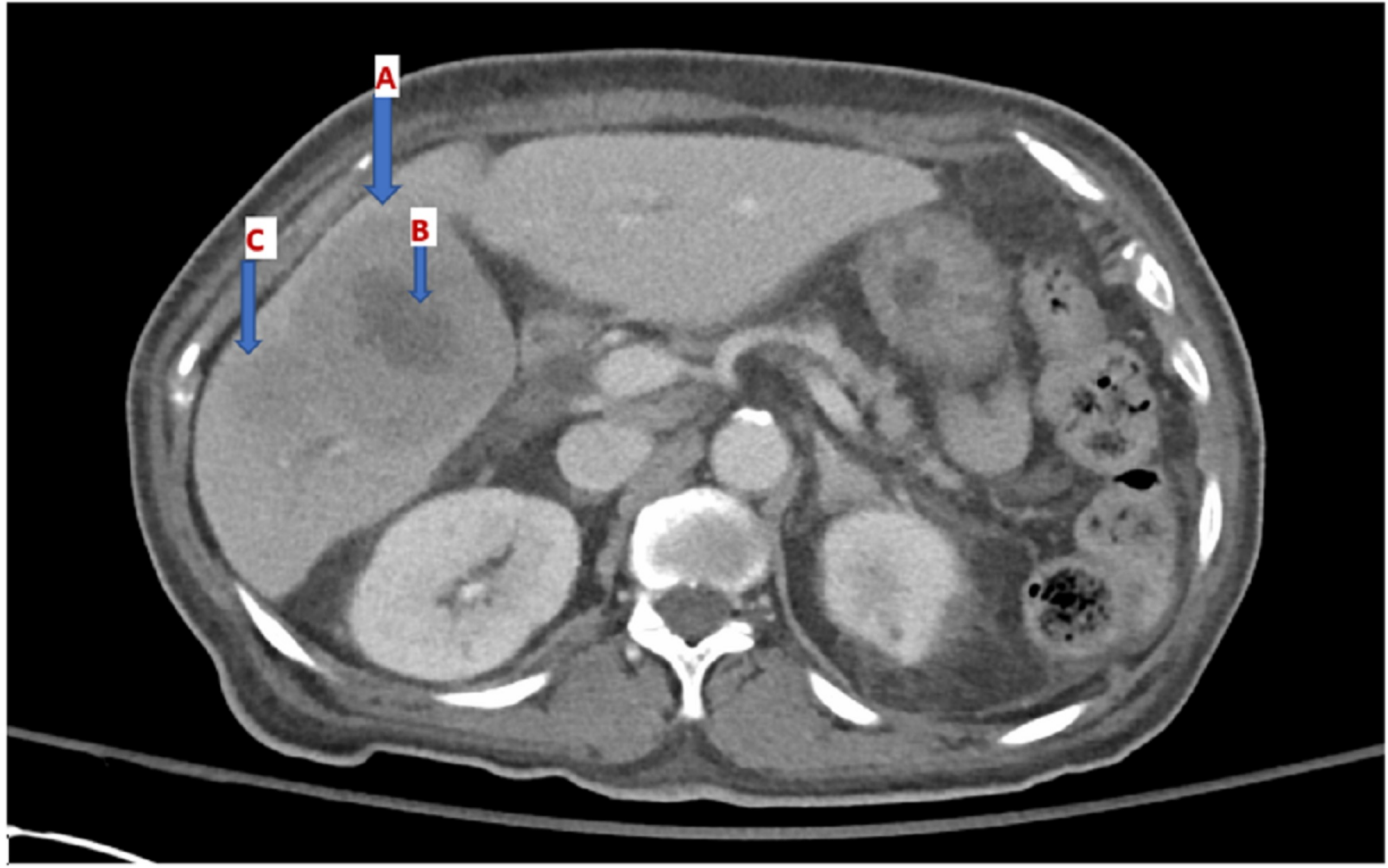
CT of the abdomen and pelvis depicting two hypoenhancing masses. Arrow A shows 8.8 × 7.1 × 5.7 cm hypoenhancing lesion in the right lobe of liver. Arrow B shows area of central necrosis. Arrow C shows 6.0 × 5.8 × 5.3 cm lesion.

CT of the abdomen was performed and showed two adjacent hypoenhancing large masses in the right hepatic lobe with central areas of hypodensity/necrosis: one at segment V measuring approximately 8.8 × 7.1 × 5.7 cm, while the other at segment VI measuring 6 × 5.8 × 5.3 cm (Figure 1). Alpha-fetoprotein (AFP) and carcinoembryonic antigen (CEA) levels were normal. Because the initial impression was concerning for hepatocellular carcinoma, a triphasic CT scan of the liver was done but provided no definitive diagnosis. Viral hepatitis serologies were also negative. A CT of the chest was unremarkable. 

**Figure 2:**
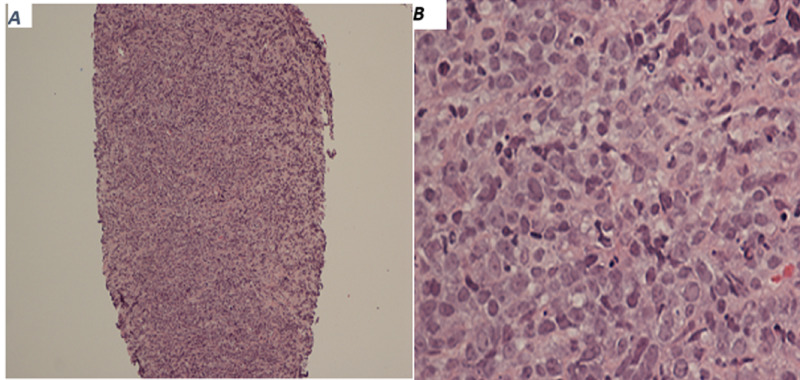
Hematoxylin and eosin staining showing diffuse proliferation of atypical lymphocytes in liver tissue at lower magnification (A) and higher magnification (B).

**Figure 3:**
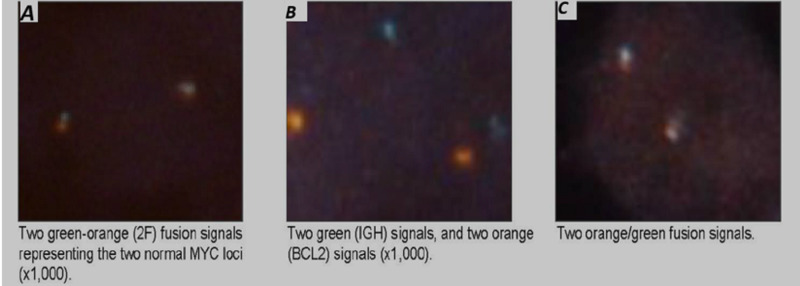
Fluorescence in situ hybridization (FISH) showing staining of MYC (A), BCL2 (B), and BCL6 (C) in lymphoma cells.

Percutaneous liver biopsy under ultrasound guidance was then pursued. Histology, immunohistochemistry (Figure 2), and fluorescent in situ hybridization (FISH) report (Figure 3) showed diffuse proliferation of atypical cells positive for CD10, BCL-2, and BCL-6 with low C-myc positivity, consistent with large B cell lymphoma. The patient was evaluated by the inpatient oncology team for chemotherapy. She was discharged five days later and is currently receiving rituxan-cyclophosphamide, hydroxydaunorubicin, vincristine, and prednisone (R-CHOP regimen).

## Discussion

PHL is defined as a lymphoma either confined to the liver or having major liver involvement [2]. PHL is very rare, representing less than 1% of all extra nodal lymphomas. Although the exact reason for its rarity is unknown, it is postulated that certain host factors make the liver an unfavorable environment for the development of malignant lymphoma. PHL is more common in men but, as in our case, can also occur in women, with a male to female ratio of 2-3:1 [3].

Various viral etiologies, particularly hepatitis C virus (HCV), have been suggested to play a role in the development of PHL [4]. Our patient hepatitis serologies including HCV were negative. Patients with PHL typically present with non-specific abdominal complaints, and some have the classic B symptoms of lymphoma, including fever, night sweats, and weight loss [5]. Often, liver function tests at presentation are abnormal.

Our case is unique as the patient presented with symptomatic hypercalcemia and almost normal liver function tests except for mildly elevated ALP. In lymphoma, tumor production of calcitriol is the main mechanism of hypercalcemia although it is unclear if hypercalcemia is a poor prognostic factor or not. In our patient, hypercalcemia responded to conventional treatment modalities.

Imaging usually shows hypoenhancing or hypoattenuating single or multiple liver masses. AFP and CEA are typically within normal range. Definitive diagnosis is made by biopsy with diffuse large B cell lymphoma being the most common type [5]. In our case, there was no evidence of splenic or lymph nodal involvement on CT abdomen and CT scan of the chest and neck showed no evidence of lymphoma.

Most patients are treated with chemotherapy. The standard being the CHOP regimen [6]. The addition of rituximab (anti-CD20 monoclonal antibody) augments the complete response rate and prolongs event-free and overall survival. Surgical resection and radiotherapy have been shown to play a role in a selected cohort of patients [6,7].

## Conclusions

PHL should be considered in a patient with a liver mass with normal levels of AFP and CEA. As opposed to other types of liver cancer, it responds to chemotherapy and has a relatively good overall prognosis and survival. Symptomatic hypercalcemia of malignancy could be the initial mode of presentation and is readily responsive to early institution of aggressive IV fluids and IV bisphosphonates therapy.
